# Evaluating a Lung Abscess in a Pediatric Patient using Point of Care Ultrasound (POCUS)

**DOI:** 10.24908/pocus.v9i2.17603

**Published:** 2024-11-15

**Authors:** Alisha Ching, Charles William Kropf

**Affiliations:** 1 Department of Emergency Medicine, University of Michigan Health System, Michigan Medicine Ann Arbor, MI USA

**Keywords:** Lung POCUS, Lung Ultrasound, Lung, Abscess, Pediatric, POCUS Case Presentation

## Abstract

Application of ultrasound to evaluate pediatric respiratory disease in the emergency department setting is rapidly growing, particularly as we often weigh the risks of exposure to radiation with other readily available imaging modalities in the acute care setting. In this case report, we describe how point of care ultrasound (POCUS) was utilized by emergency providers to characterize a lung abscess diagnosed in a pediatric patient. We also compare the ultrasound findings to other imaging studies.

## Case Presentation

A 5-year-old previously healthy, fully immunized male presented to the emergency department for evaluation of right-sided chest and abdominal pain. His parents had noted shallow breathing and a mild cough described as “throat clearing.” The patient had spiked a fever of 102˚F about one week prior but had been afebrile in the days leading up to his presentation to the emergency department. Vital signs were age-appropriate, including a normal respiratory rate of 20 and oxygen saturation of 99% on room air. On exam, the patient was noted as happy and interactive, although had shallow respirations and coarse breath sounds in the right middle lung field on auscultation. Lab results revealed a white blood cell count of 19.8 x 10^3^ cells/mL, elevated C-reactive protein of 10.1 mg/dL, and elevated erythrocyte sedimentation rate of 74 mm/h.

**Figure 1:**
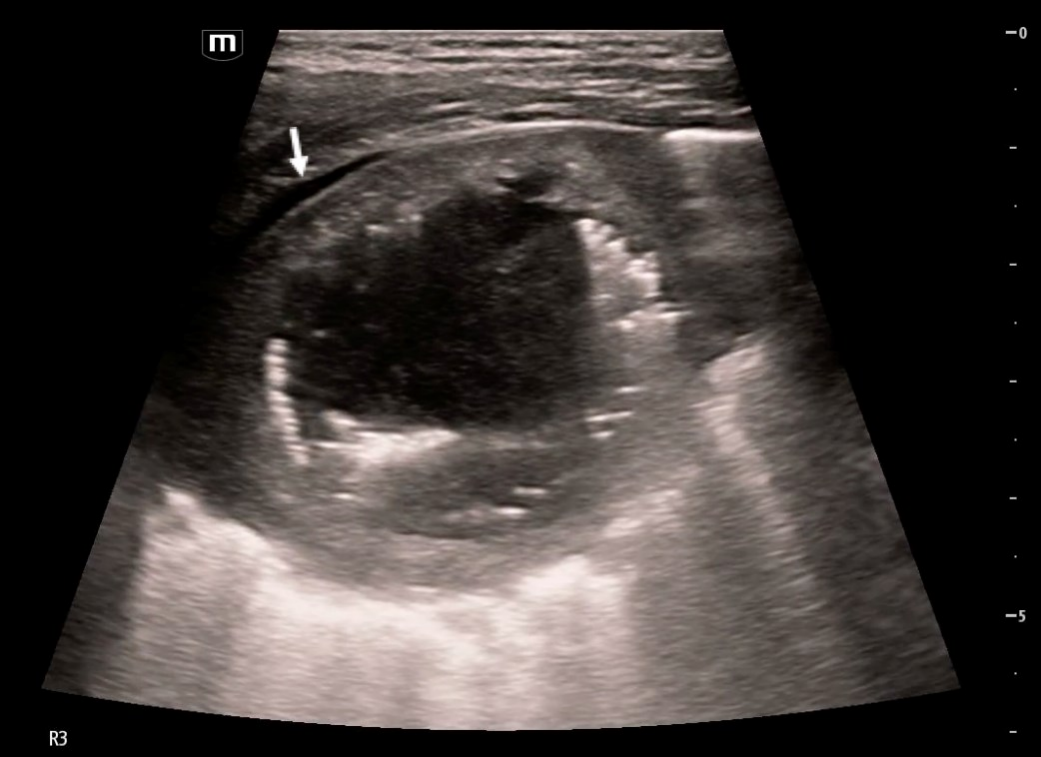
Cavitary lesion with heterogeneous internal contents with gravity-dependent layering imaged in lung zone R3 in the horizontal plane with an L-12 linear probe. An associated pleural effusion is marked in the image (white arrow).

**Figure 2:**
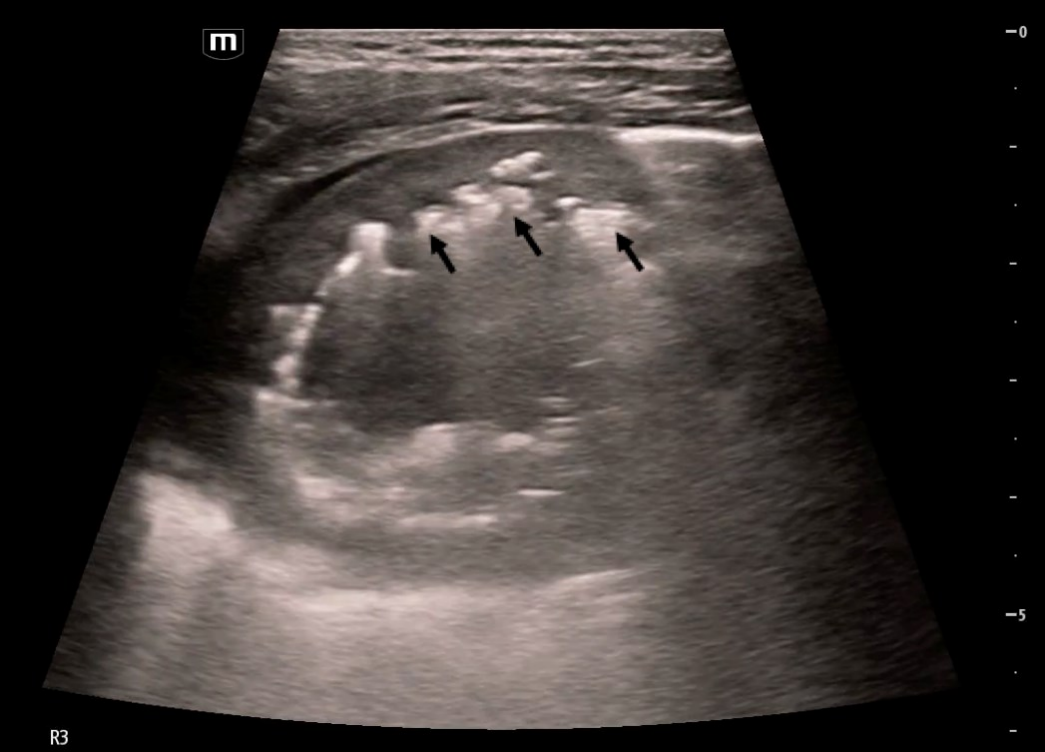
Cavitary lesion with heterogenous internal contents imaged in lung zone R3 in the horizontal plane with an L-12 linear probe. Gravity anti-dependent hyperechoic areas (black arrows) are seen within the cavity with dirty shadowing, indicative of the presence of air.

**Figure 3:**
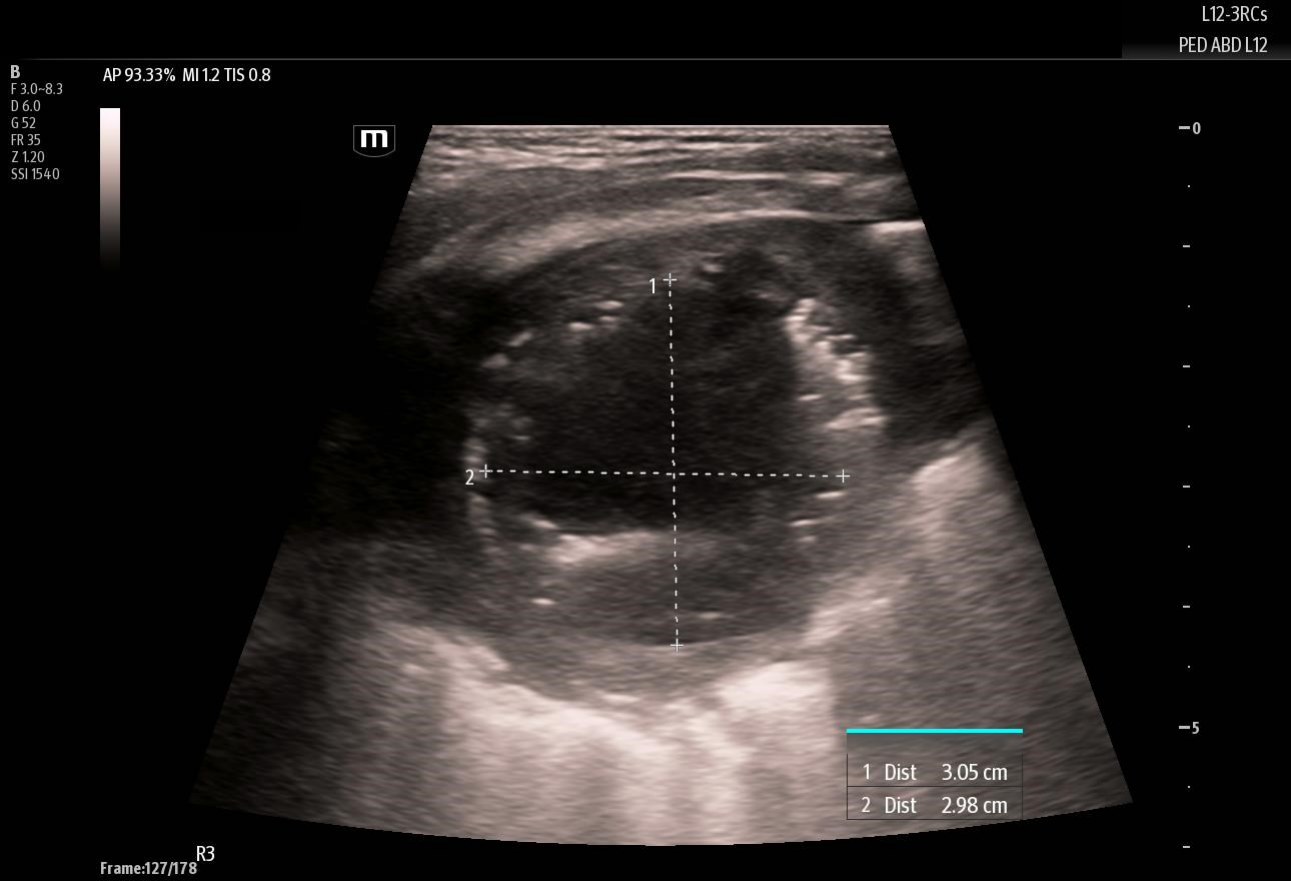
Measurements of the lung abscess internal cavity.

The patient underwent imaging evaluation, beginning with a chest radiograph that revealed a right lower lobe cavitary lesion measuring approximately 6 cm in diameter with an air-fluid level. Next, our emergency physician team decided to further characterize the lung abscess using POCUS, where we readily visualized the cavitary lung lesion in great detail. A well-circumscribed round lesion with heterogenous contents and surrounding lung consolidation was appreciated, as well as a small pleural effusion adjacent to the lesion (Figure 1), which was not visualized in the chest radiograph. Layering gravity-dependent heterogeneous contents are noted (Figure 2), including hyperechoic gravity anti-dependent contents representing air. Color Doppler revealed no vascular flow within the lesion, and measurements of the internal cavity of the lesion demonstrated a diameter of about 3 cm (Figure 3).

The patient did not have an oxygen requirement and did not meet admission criteria at this first presentation. He was discharged on a course of amoxicillin-clavulanic acid per Pediatric Infectious Disease consultation recommendations. Notably, he did not undergo a chest computed tomography (CT) scan on this first visit given the quality of POCUS in characterizing the lesion. However, he presented back to the emergency department five days later due to ongoing fevers and chest pain despite adherence to his oral antibiotic regimen. As a result, he was then admitted to the hospital for intravenous antibiotics including vancomycin and ampicillin-sulbactam. Chest radiograph demonstrated similar findings to the previous presentation, and the patient subsequently underwent a chest CT which again demonstrated the lung abscess with associated pleural effusion. Pediatric Surgery and Interventional Radiology were consulted, and the patient underwent chest tube placement to drain the pleural effusion. He was discharged home after a nine day hospitalization on an oral regimen of amoxicillin-clavulanic acid and trimethoprim-sulfamethoxazole to complete a four week course of antibiotics.

The patient presented again to the emergency department six days after discharge with new fevers up to 100.6˚F. A chest radiograph redemonstrated the lung abscess with slight interval increase in the size of the lesion by nearly 1 cm; this measurement was consistent on a follow-up radiology-based lung ultrasound. The patient was again admitted for intravenous antibiotics. Another chest CT was completed, revealing that the lesion remained a similar size in comparison to prior CT imaging, though the cavity had fewer contents within it. This interval change is consistent with natural resolution of the disease process. The patient tested positive for human rhinovirus/enterovirus during this third encounter and was able to be discharged home back to his oral antibiotic regimen of amoxicillin-clavulanic acid and trimethoprim-sulfamethoxazole after a two day hospitalization. At outpatient follow-up at the end of his four week antibiotic course, radiographs showed significant improvement in the size of the abscess, and the patient ultimately recovered completely.

### Technique

Lung POCUS can be completed with either a curvilinear or linear probe. In adult populations, curvilinear probes are often preferred due to their ability to penetrate adequately through the subcutaneous tissue and lung. In pediatric populations, higher frequency linear probes are often adequate for lung ultrasound due to the smaller size of this patient population.

In preparation for our lung POCUS, the patient was seated upright in the gurney in a comfortable position at approximately a 45˚ angle. The patient’s cavitary lung lesion was imaged with a Mindray TEX L-12 linear ultrasound transducer in lung zone R3 (upper mid-axillary line) using an extended field of view. The lesion was imaged in both the horizontal and vertical planes. Five-second clips of the lesion were saved as we fanned through the lesion to capture the full extent of the lesion. Color Doppler was applied to the lesion to assess for vascularity. Additional clips of lung zones R1, R2, and R4 were captured, as well as clips of the opposite lung zones L1 through L4 for comparison.

See Supplemental Images online to view five second clips of the lung abscess. Also included in the Supplemental Images are correlating imaging studies, including chest radiograph findings and CT findings.

### Review of the Literature

Respiratory illness is a common chief complaint for pediatric patients in the emergency department. To reduce exposure to radiation for pediatric patients, point of care ultrasound (POCUS) is being increasingly utilized in the emergency department setting as an alternative means to diagnose various respiratory conditions. Current applications for pediatric lung POCUS include evaluation of lung consolidation and pneumonia, viral processes such as bronchiolitis, pleural effusions, and trauma applications such as pneumothorax, lung contusions, and drowning 1, 2.

Chest radiographs and CT imaging are modalities often utilized in the emergency department for evaluation of progression and resolution of disease processes, such as this patient’s complicated pneumonia with abscess. Other case reports have demonstrated similar successes in identifying lung abscesses in pediatric patients using POCUS 3, 4. 

There are few studies so far examining the use of lung ultrasound for disease process monitoring for complicated pneumonia, though lung ultrasonography shows promising results in comparison to chest CT as a gold standard for monitoring of lung abscess^5^ as well as for clinical monitoring and predicting outcomes of patients with complicated pneumonia in the inpatient setting 6.It has also been established that lung ultrasonography can often be limited in its applications based on a patient’s body habitus, patient cooperation, and the location of the disease process within the lung parenchyma 1. Thus, this suggests that future research efforts should aim to evaluate whether stable pediatric patients with peripheral lung abscesses can be monitored with POCUS, as there is potential to positively impact this patient population by reducing exposure to ionizing radiation.

## Conclusions

This patient’s complicated disease course spanned three separate hospital encounters and outpatient visits. This resulted in five separate chest radiographs and two chest CT scans in about a one-month span. The patient’s lung abscess was visualized in great detail with lung POCUS on the first visit, likely due to its position within the periphery of the lung, as well as the absence of air in the surrounding consolidated parenchyma. This allowed our ultrasound waves to penetrate to the level of the lesion without interference.

This case raises the question of the effectiveness of lung POCUS as a modality to monitor this patient’s disease process to avoid further ionizing radiation. Our institution’s current model of ultrasound includes limited days of the week when an ultrasound team is available to perform POCUS. While some pediatric emergency medicine faculty are comfortable utilizing lung POCUS, this is inconsistent across all providers. This may have impacted the imaging modality that was chosen to evaluate this patient’s lung abscess across multiple presentations with different providers in the emergency department. Through ongoing POCUS education workshops with our pediatric emergency medicine providers, we hope that in the future, patients similar to the one discussed in this case could undergo repeated lung POCUS exams to evaluate their pathology to help reduce radiation exposure.

This case illustrates that peripheral lung abscess in pediatric patients can be readily evaluated with lung POCUS.

## Statement of Informed Consent

Written informed consent was obtained from the patient/guardian to utilize all images (still photos, video clips) for this publication. 

## Disclosure Statement 

The authors declare that they have no competing interests.

## Supplementary Material

**Figure S1:**
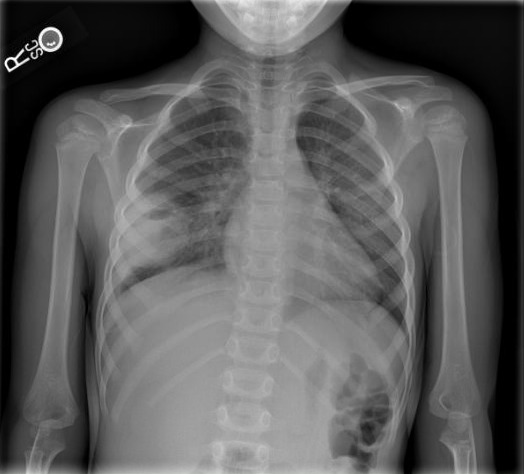
Initial Chest Radiograph.

**Figure S2:**
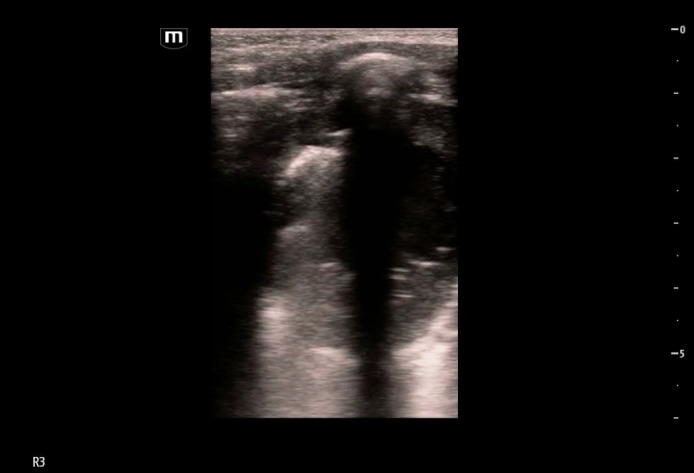
Cavitary lesion imaged in lung zone R3 in the vertical plane with an L12 linear probe. The lesion is more difficult to visualize in this plane due to the presence of rib shadows.

**Figure S3:**
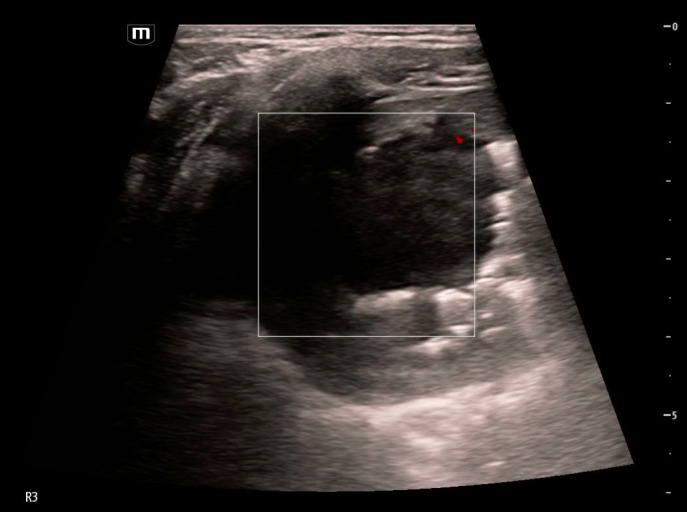
Cavitary lesion with color doppler demonstrating no vascular flow within the cavity.

**Figure S4:**
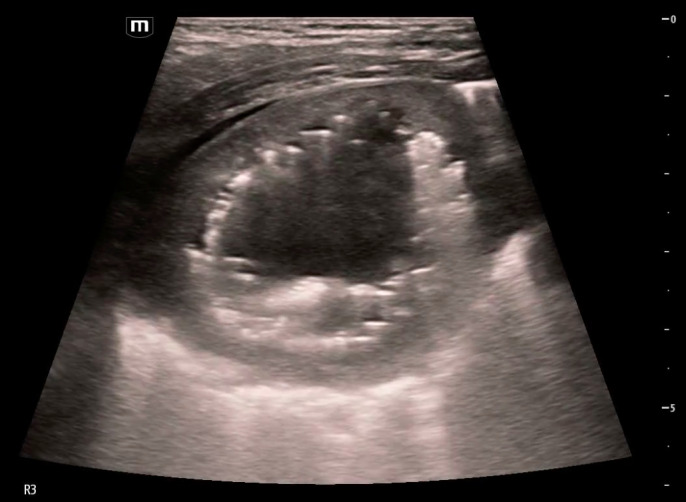
Additional demonstrations of our ability to characterize this lesion, imaged in lung zone R3 in the horizontal plane with an L-12 linear probe.

**Figure S5:**
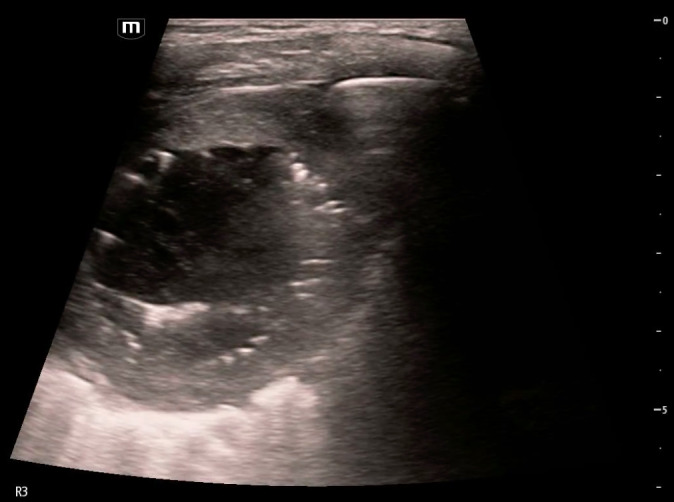
Additional demonstrations of our ability to characterize this lesion, imaged in lung zone R3 in the horizontal plane with an L-12 linear probe.

**Figure S6:**
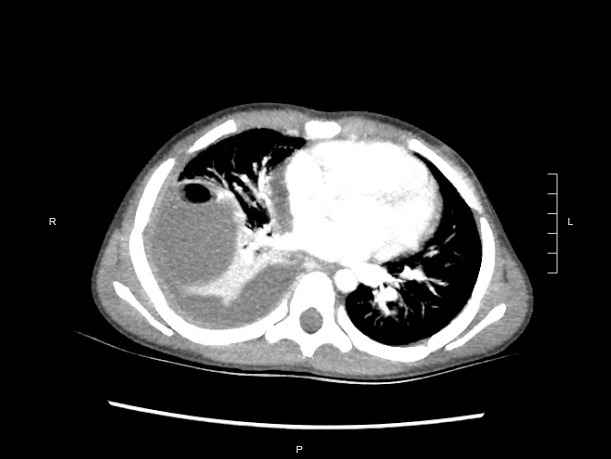
Chest CT at time of the patient’s second presentation at the hospital, demonstrating a right cavitary lung lesion with air-fluid level.

**Figure S7:**
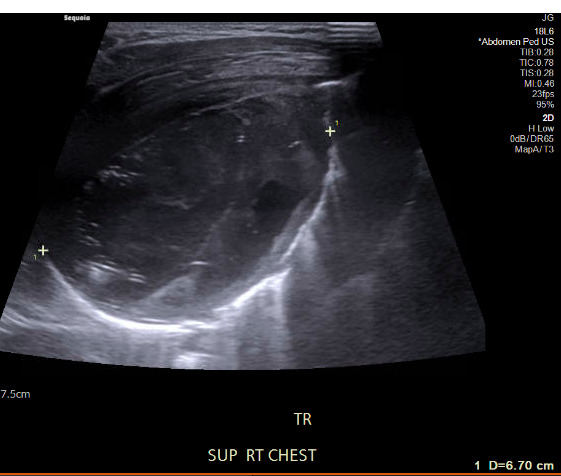
Radiology-based lung ultrasound demonstrating the lung lesion with similar measurements to the chest x-ray in the same encounter.

**Figure S8:**
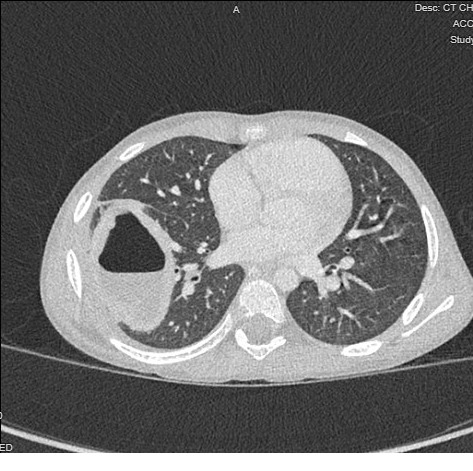
Repeat chest CT during the patient’s third hospital encounter.

 Video S9 Cavitary lesion.

Video S10 Cavitary lesion with Color and Doppler.
